# Smallpox in Texas

**Published:** 1886-01

**Authors:** 


DANIEL'S

### Medical Journal,

PUBLISHED MONTHLY AT

AUSTIN, TEXAS.

F. E. DANIEL, M. D., EDITOR AND PUBLISHER

Subscription Price: Two Dollars per Annum in Advance.

Papers for publication in this Journal should be in the hands of the Editor by the 10th of the month preceding the month in which it is wished to appear; they must be original,—never having appeared in print elsewhere, and must be contributed exclusively to this Journal.

Contributions to the department of Original Articles are cordially invited from the professiom of Texas, expecially. What is most desired is Clinical reports of cases, essays and short practical articles on any medical topic; also solicited reports of proceedings of societies, clubs, etc., and items of medical news of general interest to the profession, such as notices of appointments of physicians, removals, changes, marriages, deaths, etc.

### EDITORIAL.

### SMALLPOX IN TEXAS.

Smallpox has appeared along the Rio Grande in many places. As a rule the local health authorities have exercised due diligence to prevent its spread.

One notable exception, however, has occurred. In the town of Presidio, in Presidio county, it has prevailed for several months. About the first of December a horse race was gotten up to break the dull monotony of the place, and neighboring sportsmen were gathering there. One party saw fit to encamp in a jacal, formerly occupied by a family of Mexicans with smallpox.

As might be expected all of them now bear upon their faces sweet souvenirs of Presidio, and smallpox prevails in many places.

Four cases are reported in San Antonio, supposed to have been brought from Gregstown just below San Antonio.

The following, as *apropos* to the subject of smallpox in Texas, we take from the Journal of the American Medical Association's report of the remarks of the several delegates to the American Public Health Association at Washington :

"Dr. Swearingen, of Texas, said that in Mexico there were ten cases of smallpox to one case in Canada, yet few cases ever occur in Texas, and no epidemic of any magnitude has ever broken out in Texas. This is on account of the inspection service at El Paso."

Referring to the above, we are authorized by Dr. S. to say his remarks, or rather his meaning, has been entirely misapprehended. What the Doctor did say about smallpox and inspection was, that the health authorities of Texas had never established along the border an inspection service against smallpox in Mexico. The long line of defense would require (in order to be effective) more men to guard, and more money to pay them than had ever been entrusted to quaratine officials in this or any other State. That inspections at railroad crossings would accomplish but little good, as the people from whom contagion is most liable travel by private conveyances. Dr. S. further says that when smallpox appeared in the State, as it frequently did, that isolation of the sick, and vaccination, was the order of the day. Smallpox could not be excluded by the most rigid and expensive quarantine, but it could be suppressed anywhere and at any time, if properly managed.

LATER.—SAN ANTONIO, January 5.—Late yesterday evening another person affected with smallpox was discovered, and to-day another was found. Both were promptly removed to the pest house. To-day the young daughter of Rodriguez, the Mexican whose family formed the first cases, died. She had been delirious since her removal to the pest house. Every effort is being made to check the further spread of the disease. The fact, however, that the cases were among us before their discovery, and that many persons had access to them, furnishes good grounds for the fear that more subjects and deaths will be reported. Vaccination is actively going on.—*Statesman.*

